# Supplementary material for: 3D-printable phosphorescent woody materials
Source: Nat Commun. 2026 Mar 11;17:3796. doi: 10.1038/s41467-026-70488-y (PMC13111626; doi:10.1038/s41467-026-70488-y)
Supplement: Supplementary file 1 — Supplementary Information [file 41467_2026_70488_MOESM1_ESM.pdf]

Supplementary Information
for
3D-printable phosphorescent woody materials

Zhijun Chen[1,2], Kai Wang[1,2], Yingxiang Zhai[1,2 *], Yujie Bai[1,2], Zijing Pan[1,2], Jingyi Zhou[1,2], Min Wang[1,2], Luyao Wang[1,2], Xue Liu[1,2], Chenhui Yang[1,2], Shouxin Liu[1], Jian Li[1], Chuanling Si[3,*], Shujun Li[1,2,*], Yiqiang Wu[4,*], and Tony D. James[5,6,*]

[1]State Key Laboratory of Woody Oil Resources Utilization, Northeast Forestry University, Harbin 150040, P.R. China.

[2]Key Laboratory of Bio-based Material Science and Technology of Ministry of Education, Northeast Forestry University, Harbin 150040, P.R. China.

[3]Tianjin Key Laboratory of Pulp and Paper, Tianjin University of Science and Technology, Tianjin 300457, P. R. China.

[4] State Key Laboratory of Woody Oil Resources Utilization, Central South University of Forestry and Technology, Hunan 410004, P.R. China.

[5]Department of Chemistry, University of Bath, Bath, UK, BA2 7AY.

[6]School of Chemistry and Chemical Engineering, Henan Normal University, Xinxiang 453007, China.

[*]Corresponding authors emails: zyx1105@nefu.edu.cn (Y. Zhai); sichli@tust.edu.cn (C. Si); lishujun@nefu.edu.cn (S. Li); wuyq0506@126.com (Y. Wu); T.D.James@bath.ac.uk (T. D James).

Reagents and materials

All wood used in this article was purchased from Alibaba (Hangzhou, China). Sodium hydroxide (NaOH, >99.9%), monochloroacetic acid ($ClCH_2COOH$, 99%), ethanol (>99.8%), Carboxymethyl Cellulose (CMC, DS = 0.9, 1500-31000 Pa·s), Borax ($Na_2(B_4O_7)\cdot 10H_2O$, >99.99%), Microcrystalline cellulose (65μm, 0.26-0.31 (g/ml)) and alkali lignin were purchased from Aladdin (Shanghai, China). Deionized water was produced by Smart-RO ultrapure water system (Hitech Instruments Co., Ltd., Shanghai, China).

Characterization

Fluorescence spectra, afterglow spectra, lifetime decay curves, and quantum yield were recorded using a FLS1000 photoluminescence spectrometer (Edinburgh Instruments, Livingston, UK) equipped with a xenon lamp and a one-microsecond lamp (detector: photoelectric multiplier, 200 nm < λ < 1700 nm). Afterglow emission spectra were recorded after a 10 ms delay. Fourier transform infrared (FT-IR) spectra were recorded using a Nicolette 6700 FT-IR spectrometer (ThermoFisher Scientific, USA) from 400 cm^{-1} to 4000 cm^{-1}, using the attenuated total reflection (ATR) method. X-ray photoelectron spectroscopy (XPS) was carried out using an ESCALAB 250x X-ray photoelectron spectrometer (American Thermo Co., Ltd., Waltham, Massachusetts, USA) equipped with a monochromatic Al Kα X-ray source. XRD patterns were recorded using an X'Pert3 powder X-ray diffractometer (PANalytical B.V., Almelo, Netherlands). The fs-TA measurements (Ultrafast Systems, Helios Fire) were performed using a femtosecond regenerative amplified Ti: sapphire laser system (Coherent, Astrella) in which the amplifier was seeded with the 100 fs laser pulses from an oscillator laser system. The laser probe pulse was produced utilizing~5% of the amplified 800 nm laser pulses to generate a white-light continuum (320-650 nm) in a CaF_2 crystal, and then this probe beam was split into two parts before traversing the sample. The film was excited by a 340 nm pump laser beam. Rheological measurement of CX-Wood was conducted using a rheometer (HAAKE MARS60, Thermo Fisher

Scientific, United States) with a pair of parallel plates and a fixed gap of 1 mm. The tensile strength and compression strength were measured using a UTM2203 universal testing machine (Shenzhen SUNS Technology Stock Co. Ltd., Shenzhen, China). The sample size for tensile testing were 75 mm × 10 mm × 2 mm, the stretching speed was 20 mm/min; The sample size used for the compression test were 18 mm × 18 mm × 18 mm, the pressure was 2000 N, the compression speed was 20 mm/min. The models were printed by a Ceramic 3D Printer (Qingdao Eazao Intelligent Technology Co., Ltd, China).

Supplementary Methods

Preparation of CX-Wood with different substitution degrees

The experimental method is as follows:

Supplementary Table 1 Preparation of CX-Wood with different Degrees of Substitution (DS).

Natural wood (g)	NaOH (g)	Alkali treatment time (min)	Alkali treatment temperature (°C)	$ClCH_2COOH$ (g)	Ether-forming reaction time (min)	Ether-forming reaction temperature (°C)
**Addition amount of NaOH**						
5	**1.5**	60	35	7	90	80
5	**3**	60	35	7	90	80
5	**4.5**	60	35	7	90	80
5	**6**	60	35	7	90	80
**Different alkali treatment time**						
5	6	**20**	35	7	90	80
5	6	**40**	35	7	90	80
5	6	**60**	35	7	90	80
5	6	**80**	35	7	90	80
5	6	**100**	35	7	90	80
5	6	**120**	35	7	90	80
**Different alkali treatment temperature**						
5	6	60	**15**	7	90	80
5	6	60	**25**	7	90	80
5	6	60	**35**	7	90	80
5	6	60	**45**	7	90	80
5	6	60	**55**	7	90	80
5	6	60	**65**	7	90	80
**Different ether-forming reaction time**						
5	6	60	35	7	**30**	80
5	6	60	35	7	**60**	80
5	6	60	35	7	**90**	80
5	6	60	35	7	**120**	80
5	6	60	35	7	**150**	80
5	6	60	35	7	**180**	80
**Different ether-forming reaction temperature**						
5	6	60	35	7	90	**40**
5	6	60	35	7	90	**50**
5	6	60	35	7	90	**60**
5	6	60	35	7	90	**70**
5	6	60	35	7	90	**80**
5	6	60	35	7	90	**90**

The degree of substitution (DS) determination of CX-Wood

For the determination of DS[1], precisely weighed 0.2g CX-Wood (accurate to 0.1mg) was added to 80% ethanol-water solution (2 mL), and HCl (2 mL) was added to the suspension to convert the CX-Wood sodium salt groups to carboxylic acid groups. The suspension was stirred for 1 h, and a solid was obtained by centrifugation. The obtained solids were rinsed twice in 80% ethanol-water solution, immersed in distilled water (20 mL), and stirred. And then, NaOH solution (25 mL, 0.1 mol/L) was poured into the above solution and heated for 15 min. Finally, the mixture was titrated with 0.1 mol/L HCl, with phenolphthalein as an indicator.

The CX-Wood content (%) was calculated using the following equation:

$$\boldsymbol{CX - Wood\ content}\ (\%) = [(\boldsymbol{V_0} - \boldsymbol{V_n}) \times \boldsymbol{M} \times \mathbf{0.059} \times \mathbf{100}]/\boldsymbol{m}$$

where V_0 is the amount of HCl used to titrate the blank solution, V_n is the amount of HCl used to titrate the samples and M is the molar concentration of HCl, and m is the sample amount.

The DS of CX-Wood was calculated using the following equation:

$$\boldsymbol{DS} = (\mathbf{162} \times \boldsymbol{CX - Wood\ content}\ (\%))/[\mathbf{5900} - (\mathbf{58} \times \boldsymbol{CX - Wood\ content}\ (\%))]$$

where the value of 162 g/mol is the molar mass of the cellulose AGU, and 58 g/mol is the molar mass increase for CX-Wood group substitution per hydroxyl group.

Life cycle assessment (LCA) methodology

This study employed Simapro 9.3 software to develop LCA models for three ink production routes (CX-ink, SI-ink, and PALF-ink) utilizing wood as a primary feedstock. The analysis was conducted based on the TRACI 2.2 methodology, with a functional unit defined as 1.0 kg of ink produced. The system boundary was set to "cradle-to-gate," encompassing raw material acquisition, energy production, chemical utilization, and wastewater treatment, while excluding infrastructure-related impacts. A process-tree modeling approach was adopted, revealing significant differences in material inputs among the routes: CX-ink consumed 210 g of wood, 252 g of sodium hydroxide, and 856 g of ethanol, with an electricity demand of 10.9 kWh; SI-ink utilized

300 g of wood and 1894 g of ethanol, requiring 29.7 kWh of electricity; whereas PALF-ink employed 300 g of wood and 1237 g of lactic acid, with an electricity consumption of 18.6 kWh. The characterization covered ten impact categories: ozone depletion (kg CFC-11 eq), global warming potential (kg CO_2 eq), smog formation potential (kg O_3 eq), acidification potential (kg SO_2 eq), human carcinogenic toxicity (CTUh), human non-carcinogenic toxicity (CTUh), fine particulate matter formation potential (kg PM2.5 eq), ecotoxicity (CTUe), freshwater eutrophication potential (kg P eq), and marine eutrophication potential (kg N eq). Data quality was assured using the Ecoinvent 3.0 database, with mass-based allocation applied and regional market averages adopted for background data such as electricity.

Preparation of CX-Wood/RhB

1.5 g of CX-Wood was mixed with 1 mL Rhodamine B solution (10 mg/mL). After adding 3 ml deionized water and thoroughly stirring, the mixture was dried at 80 °C for 4 h to obtain CX-Wood/RhB.

Preparation of CX-Wood/Polyurethane

CX-Wood (100 g, 60 mesh) was added to 120 mL of waterborne polyurethane (solid content ≥35%), followed by mechanical stirring at room temperature for 30 minutes to obtain CX-Wood/Polyurethane ink. Then, the required shape was printed using a printer. The printed structures were dried at 80 °C for 6 h to produce CX-Wood/Polyurethane printed samples.

Density functional theory

All quantum chemical calculations were performed using the Gaussian 16 software package[2]. The Becke, three-parameter, Lee–Yang–Parr (B3LYP) hybrid functional[3] was employed throughout, in conjunction with the D3(BJ) dispersion correction[4] to account for long-range van der Waals interactions. For all geometry optimizations, the def2-SVP basis set[5] was chosen, which offers a good balance between computational cost and accuracy for systems of moderate size. The independent gradient model (IGM) wavefunction analysis was carried out using the Multiwfn program[6]. Upon completion

of the optimizations, the resulting molecular geometries and relevant properties were visualized and analyzed using Visual Molecular Dynamics (VMD) software[7]. Spin-orbit coupling (SOC) matrix elements between S_1 and T_1 were computed with ORCA[8] at the PBE0/def2-SVP[9, 10] level of theory.

Additionally, to better reflect the true intermolecular interactions in the model system, a periodic model containing 20 molecules was constructed and fully optimized using the CP2K package[11] at the GFN1-xTB[12] level with variable-cell relaxation to allow both lattice parameters and atomic positions to relax. Based on the optimized structure, single-point energy calculations were subsequently performed within the framework of density functional theory using the PBE exchange-correlation functional[13] in combination with the DZVP-MOLOPT-SR-GTH pseudopotential basis set[14]. Furthermore, intermolecular interactions were analyzed and visualized using the Independent Gradient Model (IGM)[15] approach as implemented in the Multiwfn program[16].

The binding energy is calculated using the following formula:

$$E_{bind} = E_{\text{complex}} + (E_A - E_B)$$

Where E_{complex} is the total energy of the complex, and E_A, E_B are the total energies of the individual components calculated separately.

Thermo-gravimetric analysis

Samples (10 mg) were placed in the chamber of a thermal gravimetric analyzer (STA 449 F3 Jupiter, Netzsch, Germany). The test was performed in an N_2 atmosphere to simulate the thermo-oxidative decomposition process arising in the cone calorimeter, with a purge gas flow of 20 mL min^{-1}. The temperature was ramped from room temperature (approx. 30 ºC) to 800 ºC at a heating rate of 10 ºC min^{-1}. Measurements were repeated in triplicate for each treatment. The curves of residual mass (RM) and its derivative (DTG) were recorded through the tests.

Limiting oxygen index

The limiting oxygen index (LOI) of each wood sample (100 mm × 6.5 mm × 3.2 mm

(L × R × T)) was determined using an oxygen index tester (JF-3, Jiangning Analytical Instrument Co., ltd., Nanjing, China) in accordance with ASTM D2863-2017. The gas sources were O_2 and N_2 with a purity of 99.5 %.

Vertical burning tests

The vertical burning tests were determined using a CZF-2 instrument (Jiangning, China) according to ASTM D3801-1996 standard.

Cone calorimetric analysis

CC (Cone calorimetry) tests were carried out using a cone calorimeter tester (West Sussex, UK) under the heat flux of 50 $kW \cdot m^{-2}$ according to ISO 5660 standard. The dimension of specimens was 100 mm × 100 mm × 3 mm.

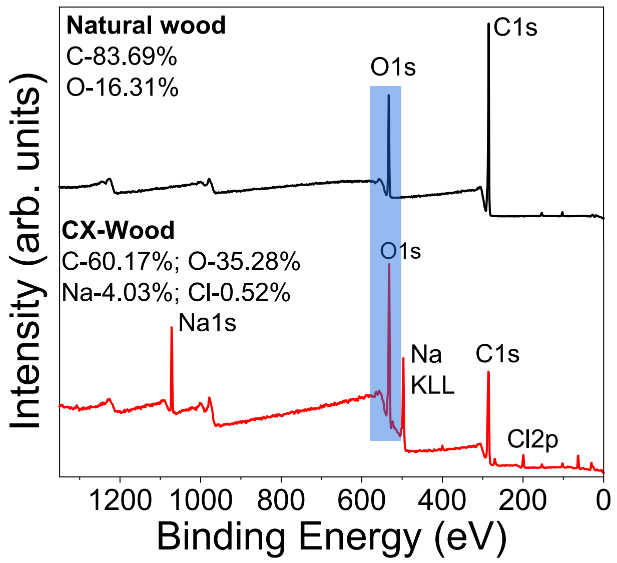

Supplementary Fig. 1. Full scan XPS spectrum of nature wood and CX-Wood.

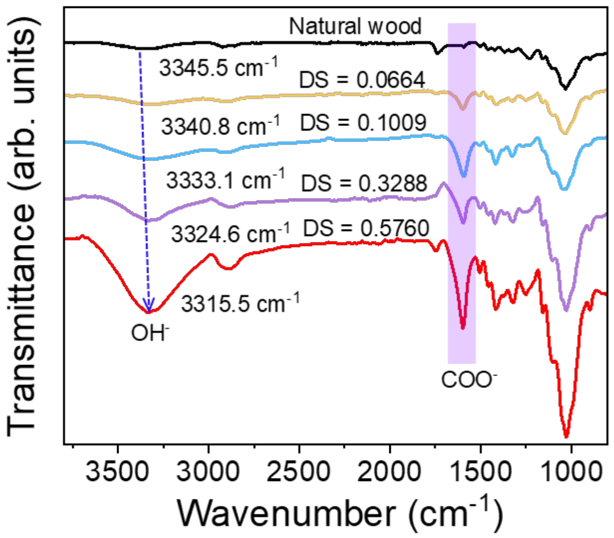

Supplementary Fig. 2. FT-IR spectra of natural wood and different substituted degree CX-Wood.

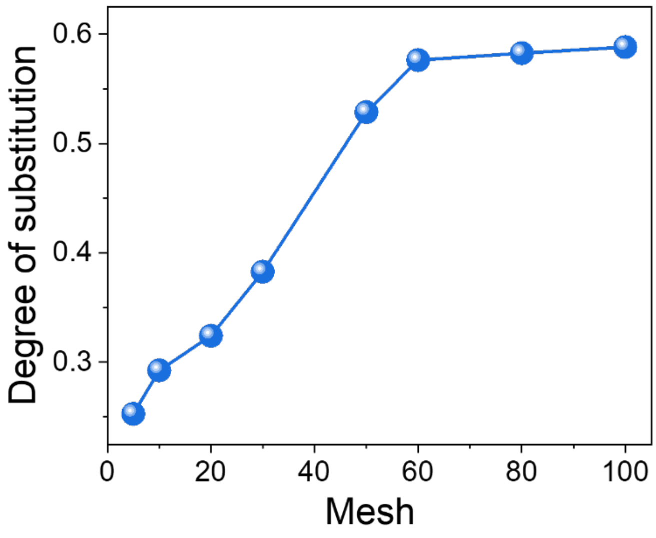

Supplementary Fig. 3. The DS of CX-Wood modified by wood powder particles of different mesh sizes.

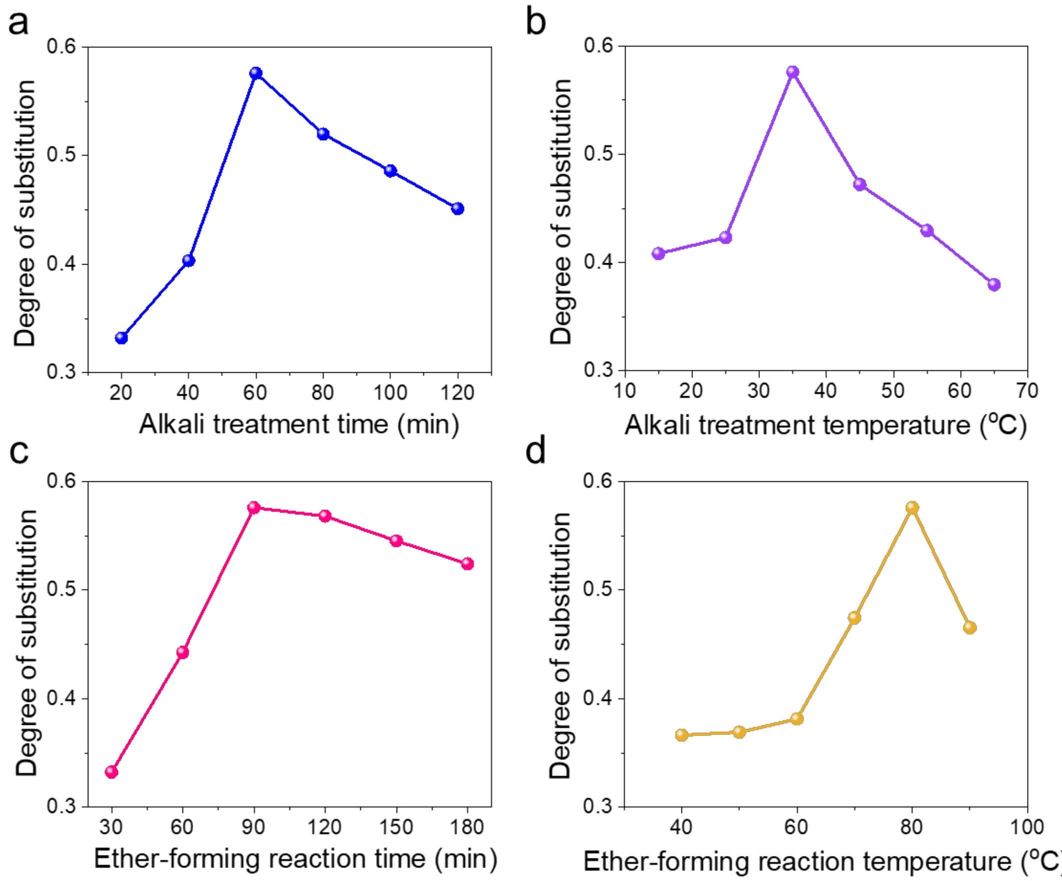

Supplementary Fig. 4. Effect of reaction time and temperature on the DS of CX-Wood. (a) Alkali treatment time. (b) Alkali treatment temperature. (c) Ether-forming reaction time. (d) Ether-forming reaction temperature.

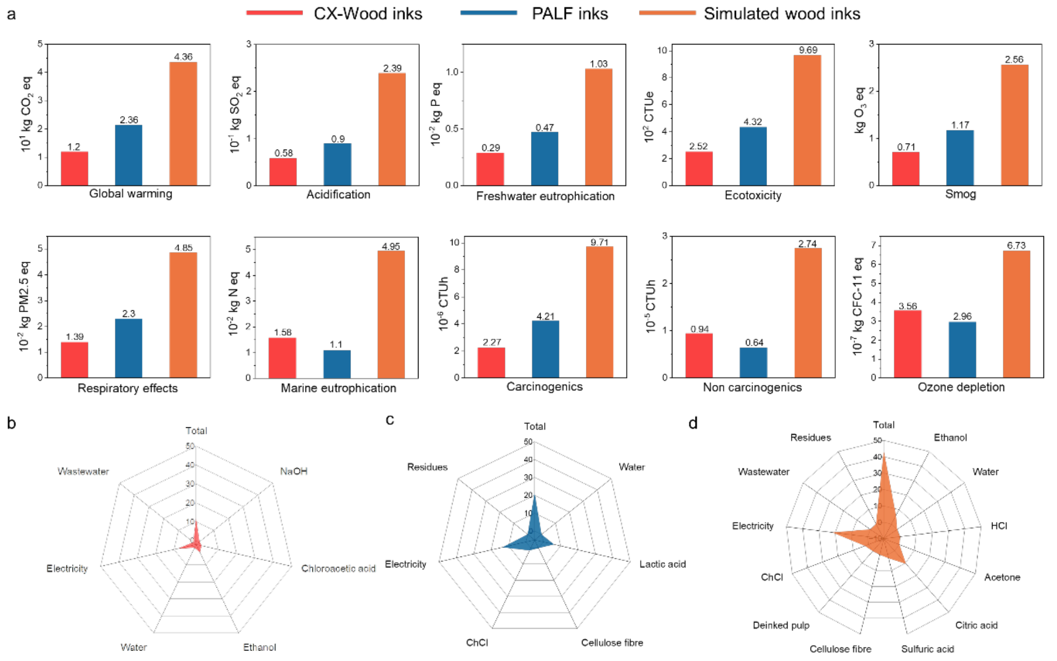

Supplementary Fig. 5. (a) Comparison of life cycle assessment results for the three inks (The data are from single calculation); (b-d) Radar charts illustrating the contributions of different materials and energy inputs to the global warming potential (GWP) indicator (functional unit: production of 1.0 kg of ink).

Supplementary Table 2 Phosphorescence lifetime vs. price: three "wood-based inks".

Types of Ink	Materials	RTP lifetime (ms)	Price ($/kg)	Ref
CX-Wood inks	Balsawood; NaOH; Chloroacetic acid	358.72	22.5	This work
Simulated wood inks[17]	Cellulose; nanocrystals; Cellulose; Nanofibers; Organosolv lignin	28.97	319	Science Advances 2024, 10, eadk3250 (Supplementary References 17)
PALF inks[18]	Lignocellulose; L-Lactic Acid; Choline chloride	72.74	53	Chemical Engineering Journal 2025, 523,168671 (Supplementary References 18)

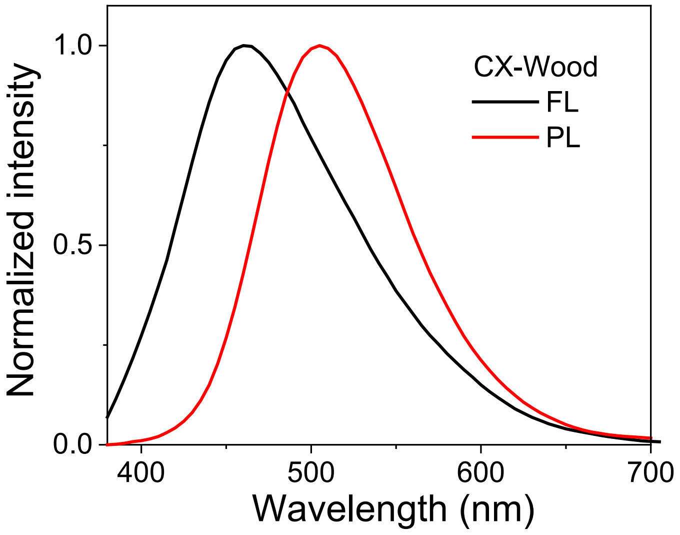

Supplementary Fig. 6. Fluorescence and Phosphorescence spectra of CX-Wood.

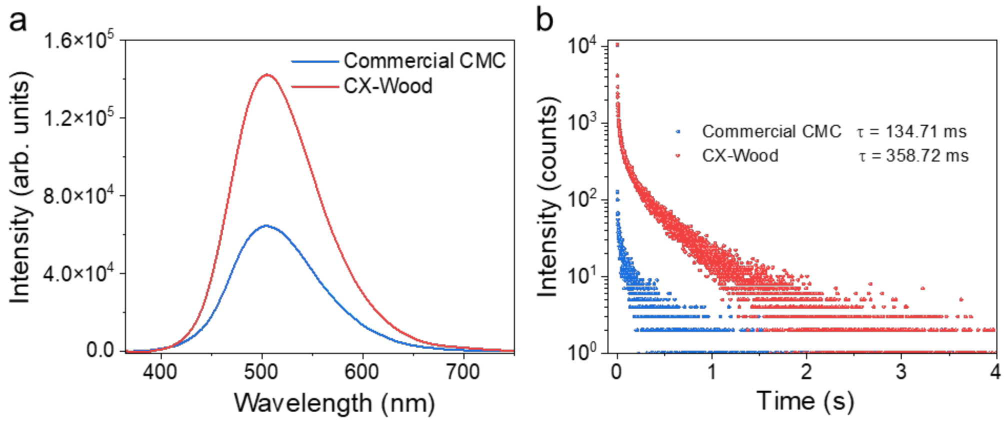

Supplementary Fig. 7. (a) Afterglow emission spectra of commercial CMC and CX-Wood. (b) Lifetime decay profiles of commercial CMC and CX-Wood. Excitation wavelength = 340 nm.

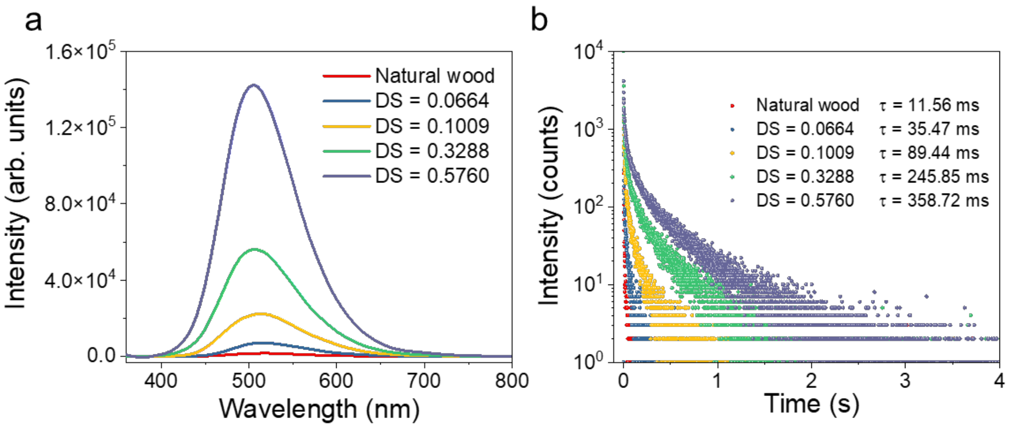

Supplementary Fig. 8. Phosphorescence properties of CX-Wood with different substitution degree. (a) Afterglow emission spectra of C-wood determined with different substitution degree. (b) Lifetime decay profiles of C-wood determined with different substitution degree. Excitation wavelength = 340 nm.

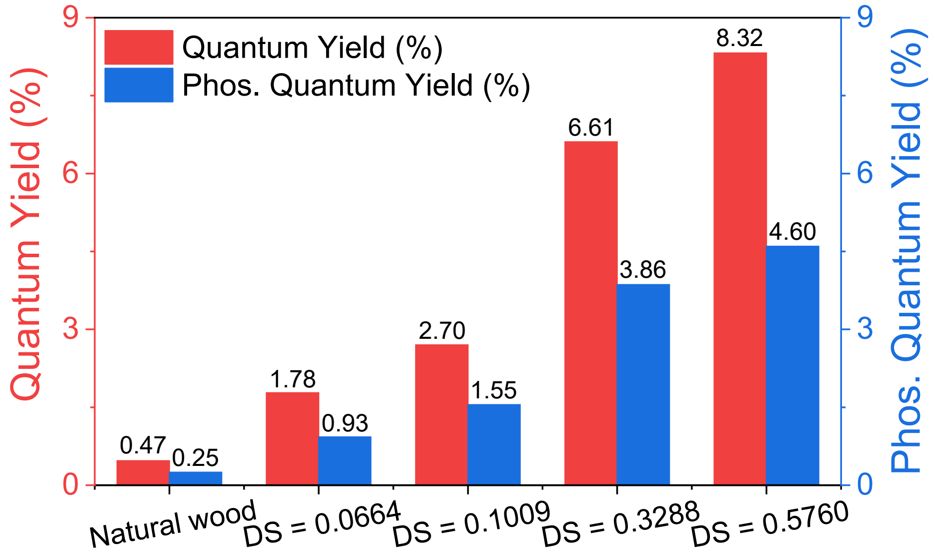

Supplementary Fig. 9. The quantum yields and phosphorescence quantum yields of natural wood and CX-Wood with varying degrees of substitution (The data are from single measurement).

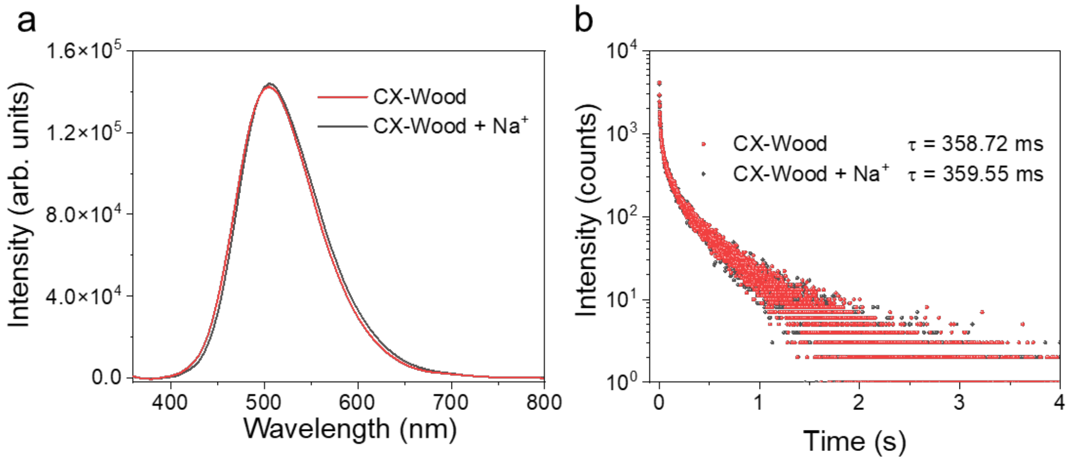

Supplementary Fig. 10. (a) Phosphorescence spectra of CX-Wood and CX-Wood + Na^+. (b) RTP decay profiles of CX-Wood and CX-Wood + Na^+, excitation wavelength = 340 nm.

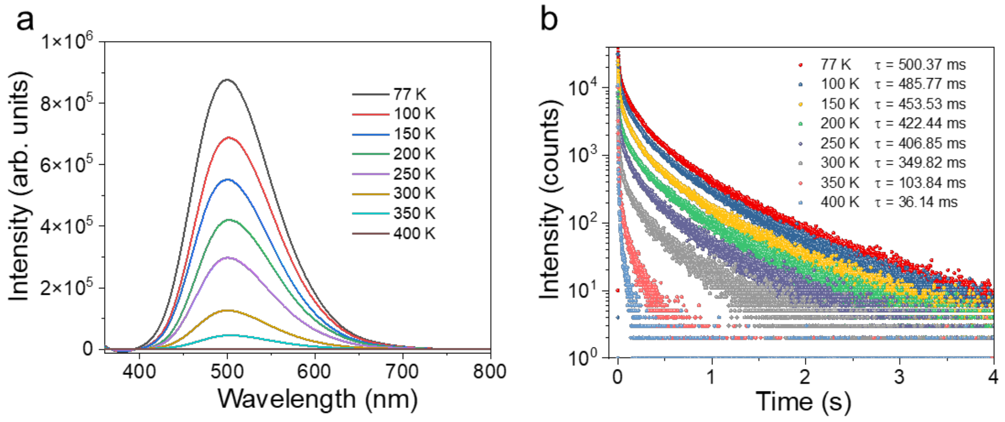

Supplementary Fig. 11. Phosphorescence properties of CX-Wood under conditions of different temperature. (a) Afterglow emission spectra of CX-Wood at different temperature. (b) Lifetime decay profiles of CX-Wood at different temperature. Excitation wavelength = 340 nm.

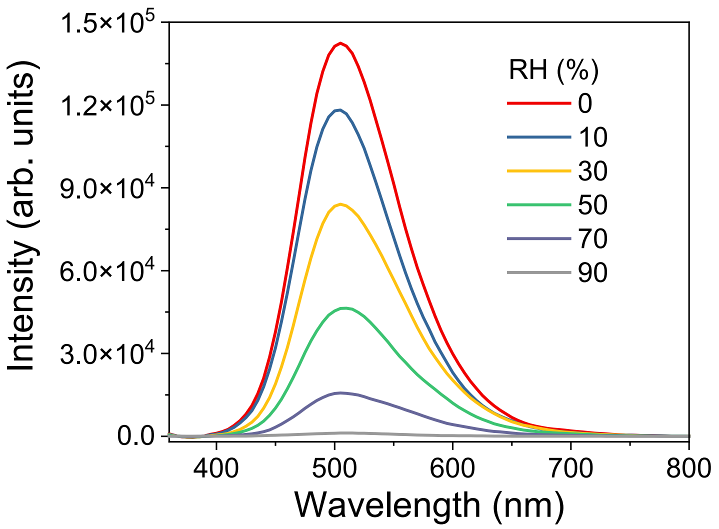

Supplementary Fig. 12. Afterglow emission spectra of CX-Wood determined under different relative humidity conditions. Excitation wavelength = 340 nm.

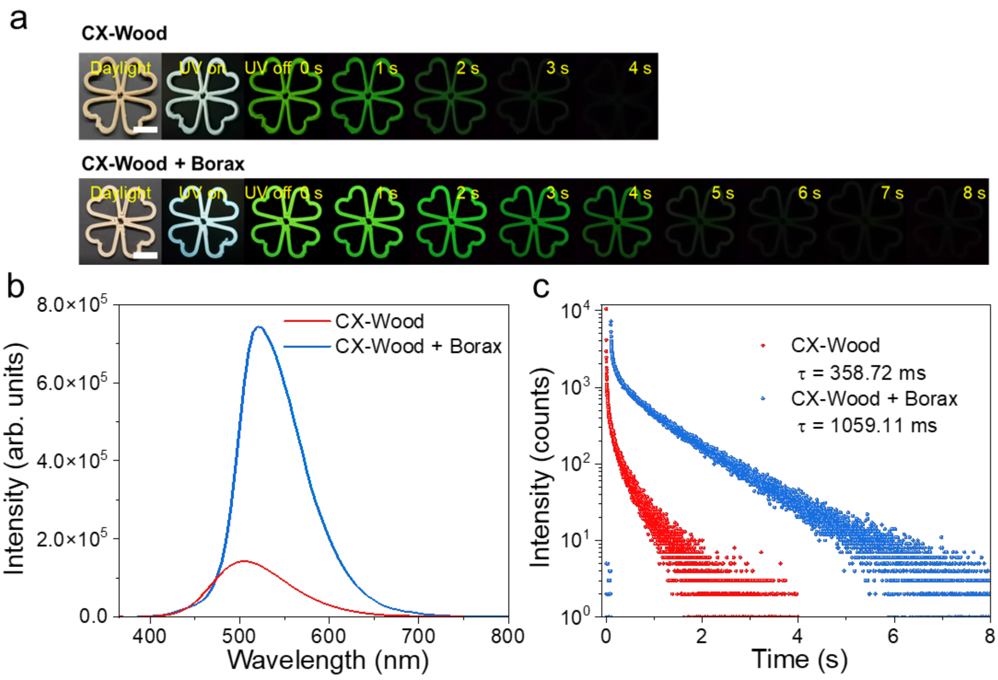

Supplementary Fig. 13. (a) Photographs of four-leaf clover printed by CX-Wood and CX-Wood + Borax after turning off a 365 nm UV lamp, scale bar = 2 cm. (b) Afterglow emission spectra of CX-Wood and CX-Wood + Borax. (c) RTP decay profiles of CX-Wood and CX-Wood + Borax. (Preparation of CX-Wood + Borax: A 200 mg/mL borax solution was prepared. CX-Wood was then mixed with the borax solution to achieve 75% solid content. The mixture was dried at 80°C to obtain CX-Wood + Borax.)

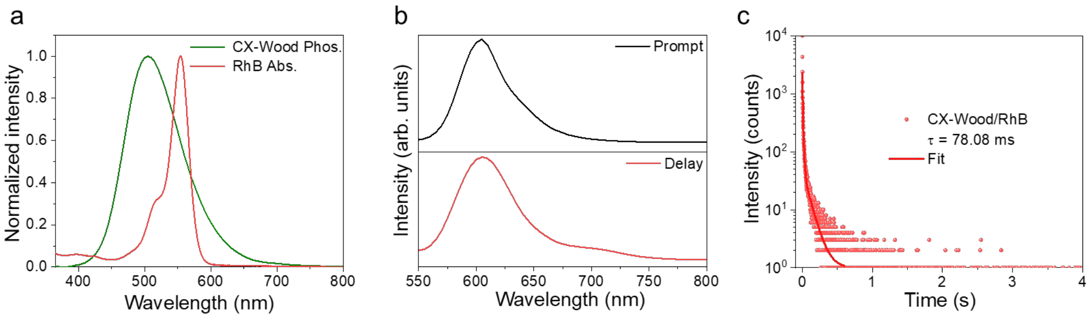

Supplementary Fig. 14. (a) The RTP emission of CX-Wood/RhB and the absorbance of RhB. (b) Fluorescence (prompt) and phosphorescence (delay) spectra of B-film/RhB. (c) Lifetime decay of delayed fluorescence of B-film/RhB at 605 nm. Excitation wavelength = 340 nm.

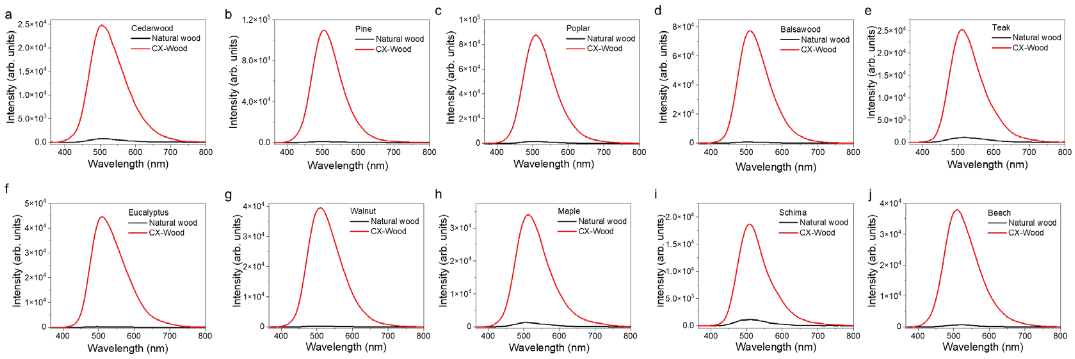

Supplementary Fig. 15. Phosphorescence spectra of different types of natural wood and CX-Wood at room temperature. (a, cedarwood; b, pine; c, poplar; d, balsawood; e, teak; f, eucalyptus; g, walnut; h, maple; i, schima; j, beech. Excitation wavelength = 340 nm.

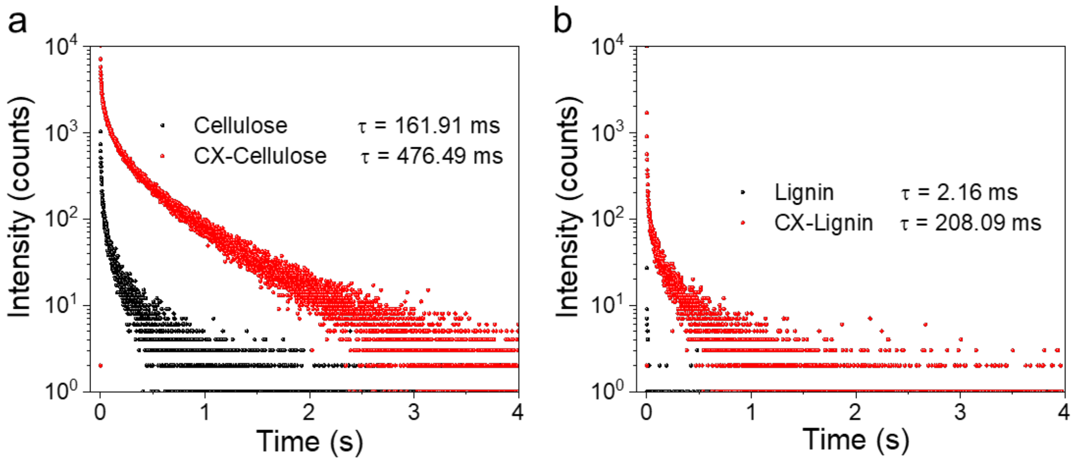

Supplementary Fig. 16. (a) Lifetime decay profiles of cellulose and CX-Cellulose. (b) Lifetime decay profiles of lignin and CX-Lignin.

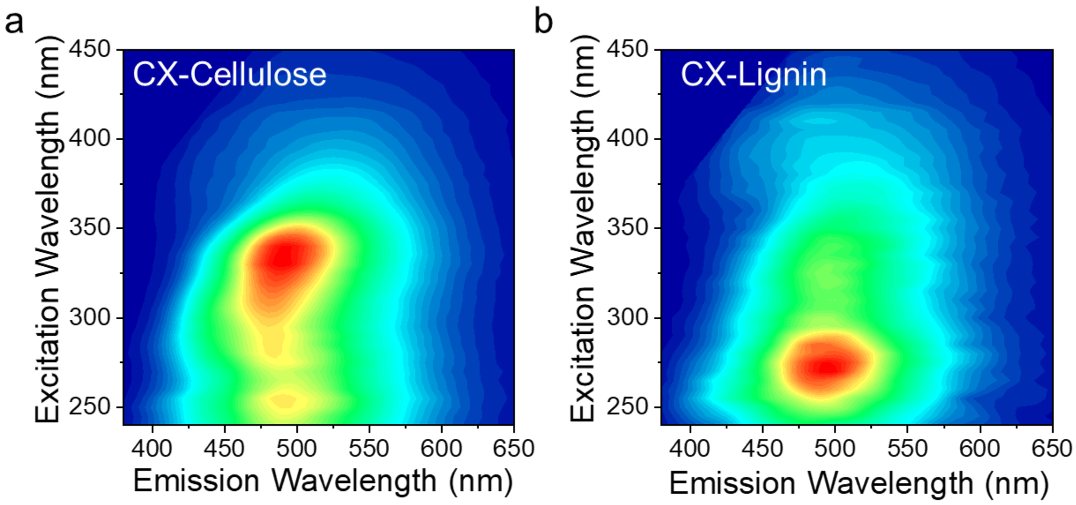

Supplementary Fig. 17. (a) RTP emissions of CX-Cellulose using different excitation wavelengths. (b) RTP emissions of CX-Lignin using different excitation wavelengths.

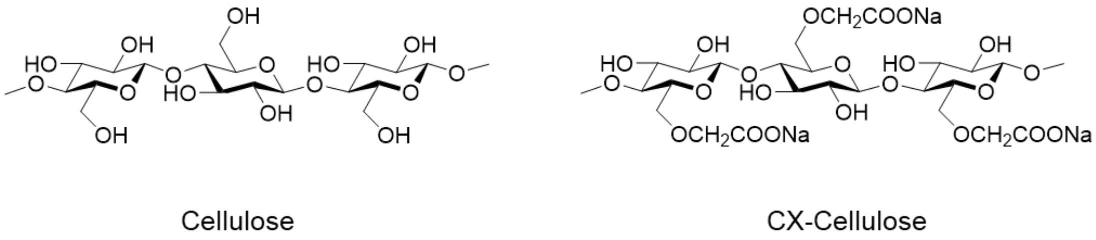

Supplementary Fig. 18. The model structures of Cellulose and CX-Cellulose used for calculations.

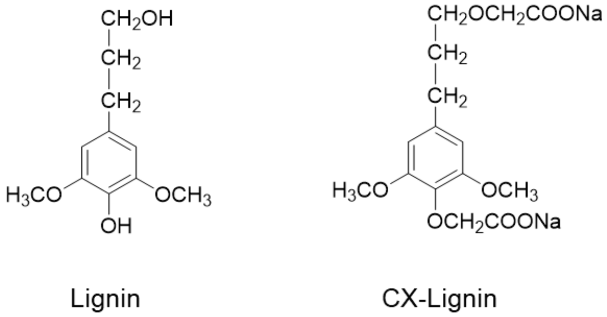

Supplementary Fig. 19. The model structures of Lignin and CX-Lignin used for calculations.

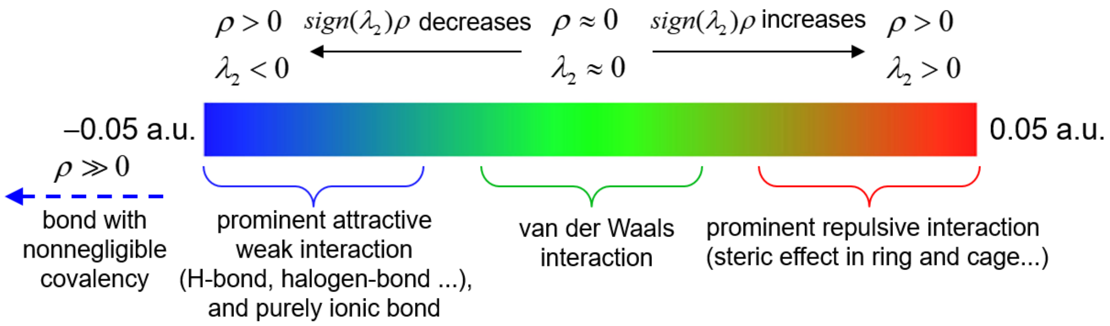

Supplementary Fig. 20. The independent gradient model (IGM) analysis. In the IGM plot, regions with darker colors signify stronger interactions. Specifically, the blue and voluminous regions represent Coulombic interactions, while the green and flat regions denote dispersion interactions.

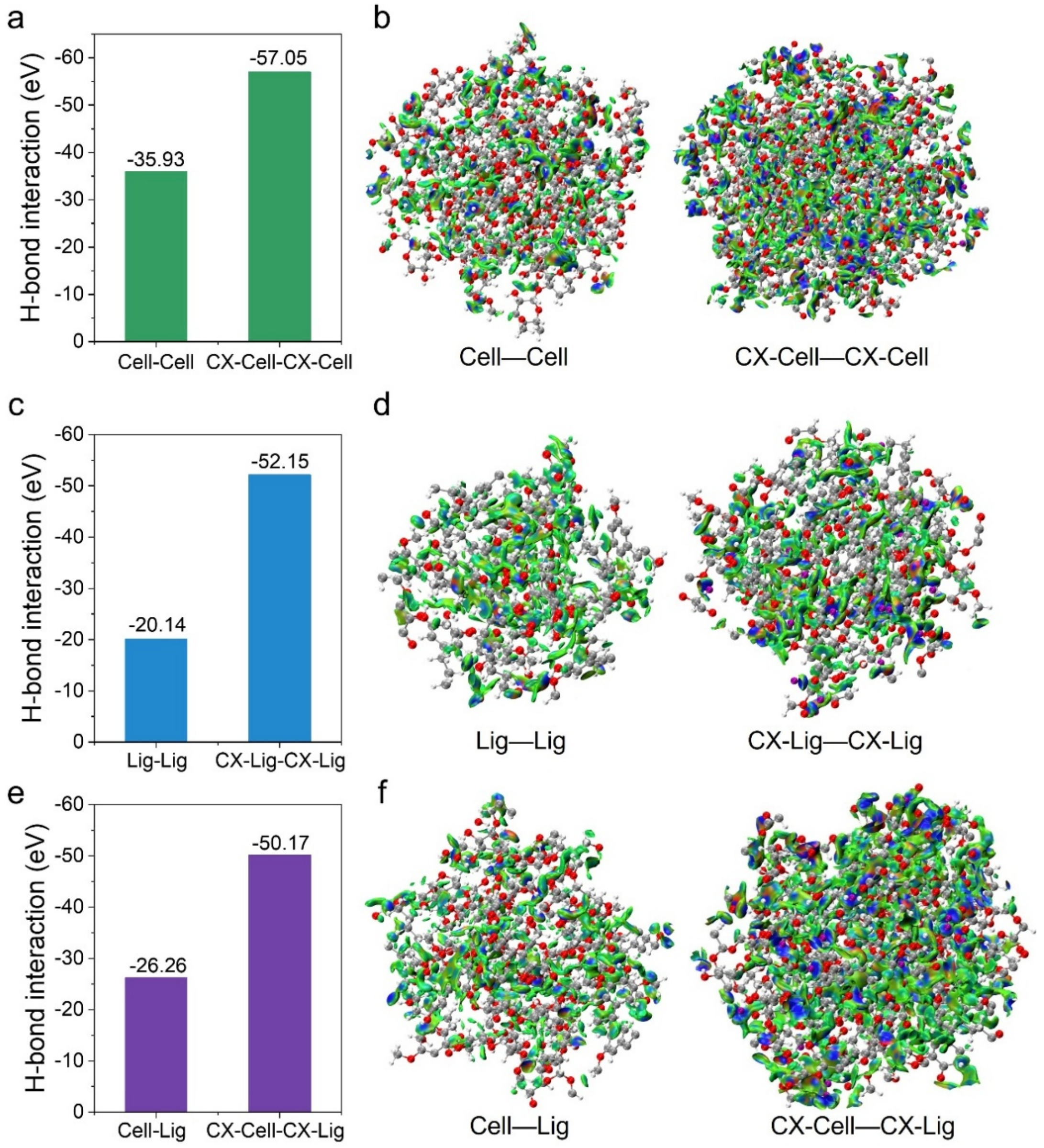

Supplementary Fig. 21. Calculations based on the periodic model containing 20 molecules. (a) Calculated interaction and (b) independent gradient models of Cellulose—Cellulose (Cell—Cell) and CX-Cellulose—CX-Cellulose (CX-Cell—CX-Cell); (c) Calculated interaction and (d) independent gradient models of Lignin—Lignin (Lig—Lig) and CX-Lignin—CX-Lignin (CX-Lig—CX-Lig). (e) Calculated interaction and (f) independent gradient models of Cellulose—Lignin (Cell—Lig) and CX- Cellulose—CX-Lignin (CX-Cell—CX-Lig).

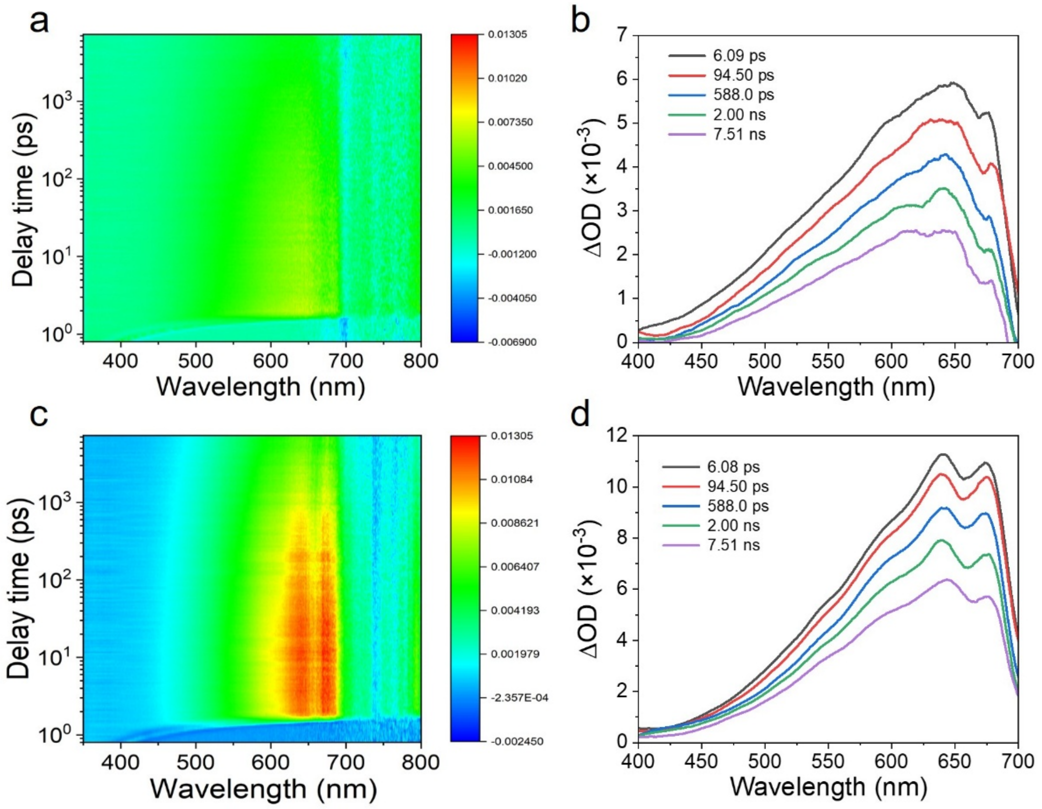

Supplementary Fig. 22. (a) Transient absorption two-dimensional temporal evolution spectra at room temperature of CX-Wood with DS = 0.3288. (b) Fs-TA spectra of CX-Wood with DS = 0.3288 under 340 nm laser excitation. (c) Transient absorption two-dimensional temporal evolution spectra at room temperature of CX-Wood with DS = 0.5760. (d) Fs-TA spectra of CX-Wood with DS = 0.5760 under 340 nm laser excitation.

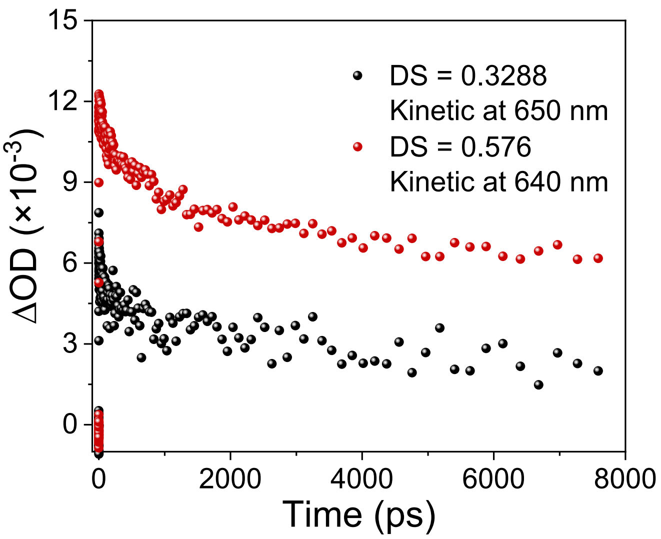

Supplementary Fig. 23. Transient absorption traces of CX-Wood with DS = 0.3288 at 650 nm and CX-Wood with DS = 0.5760 at 640 nm.

Supplementary Table 3 Photophysical properties of Natural wood and CX-Wood.

	DS	τ_F (ns)	τ_{RTP} (ms)	Φ_{RTP} (%)	$^{a)}k_{ISC}$ (s^{-1})	$^{b)}k_{nr}^{Phos}$ (s^{-1})
Natural wood	0	3.59	11.56	0.25	6.96×10^5	86.29
CX-Wood	0.0664	3.83	35.47	0.93	2.43×10^6	27.93
	0.1009	4.43	89.44	1.55	3.50×10^6	11.01
	0.3288	5.00	245.85	3.86	7.72×10^6	3.91
	0.5760	5.43	358.72	4.60	8.47×10^6	2.66

a) $k_{ISC} = \Phi_{RTP}/\tau_F$; b) $k_{nr}^{Phos} = (1 - \Phi_{RTP})/\tau_{RTP}$[19].

Supplementary Table 4 The singlet-triplet energy gap (ΔE_{ST}) of Natural wood and CX-Wood.

	DS	$\lambda_{fluo.}$ (nm)	$^{a)}E_{S1}$ (eV)	$\lambda_{phos.}$ (nm)	$^{b)}E_{T1}$ (eV)	$^{c)}\Delta E_{ST}$ (eV)
Natural wood	0	410	3.024	515	2.408	0.616
CX-Wood	0.0664	445	2.787	513	2.417	0.370
	0.1009	450	2.756	510	2.431	0.325
	0.3288	455	2.725	508	2.441	0.284
	0.5760	460	2.696	505	2.455	0.241

a) $E_{S1} = 1240/\lambda_{fluo.}$; b) $E_{T1} = 1240/\lambda_{phos.}$; c)$\Delta E_{ST} = E_{S1} - E_{T1}$[20].

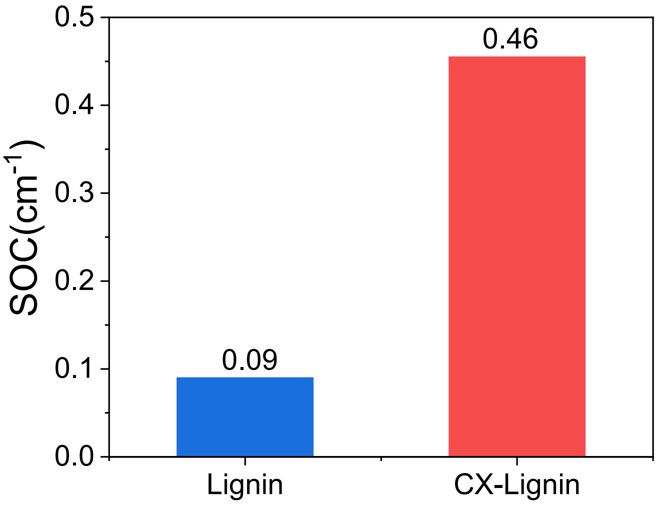

Supplementary Fig. 24. Spin orbit coupling (SOC) values between the S_1 and T_1 states calculated for Lignin and CX-Lignin (The data are from single measurement).

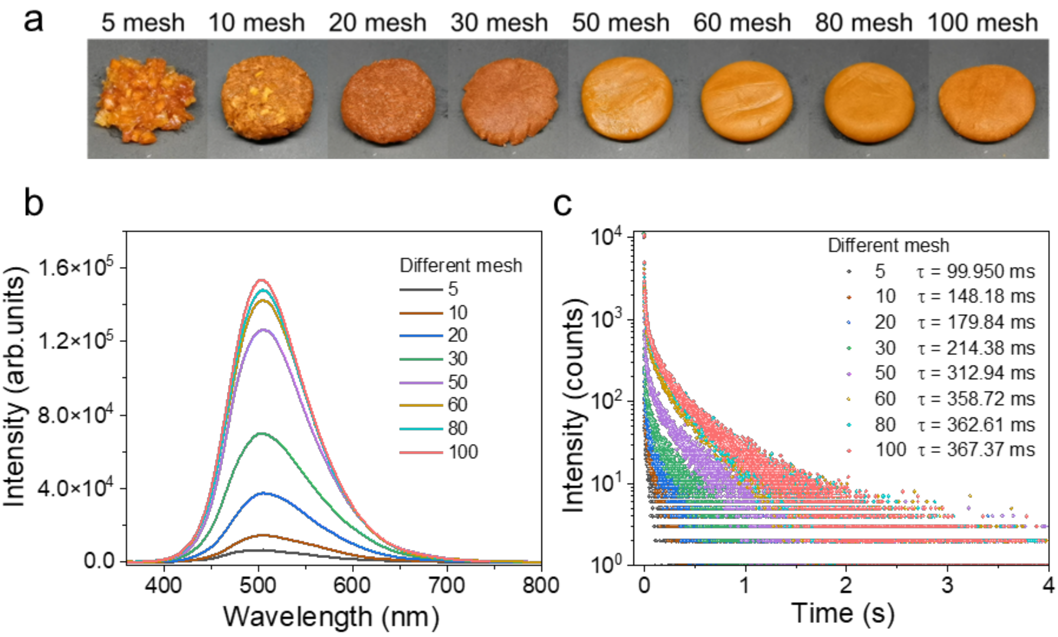

Supplementary Fig. 25. (a) The images of CX-Wood ink obtained after the modification of wood powder particles with different mesh sizes. (b) Phosphorescence spectra of CX-Wood modified by wood powder particles of different mesh sizes. (c) RTP decay profiles of CX-Wood modified by wood powder particles of different mesh sizes. Excitation wavelength = 340 nm.

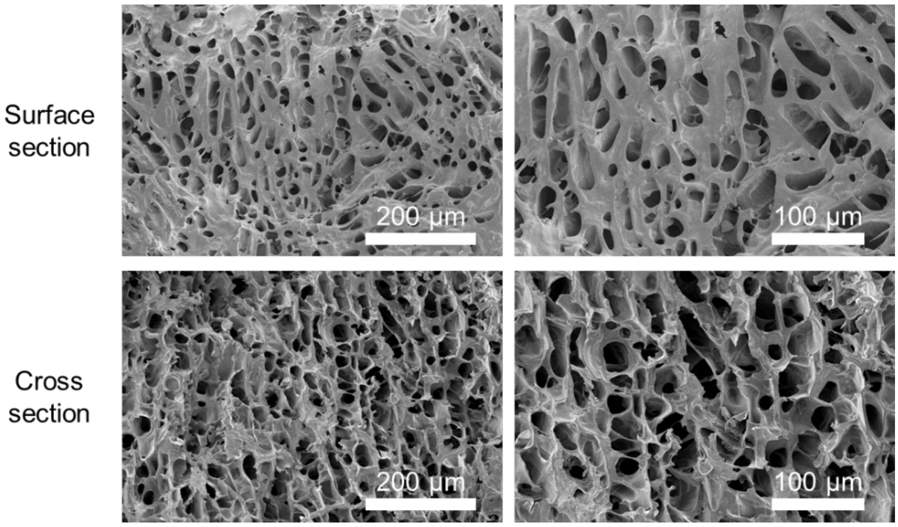

Supplementary Fig. 26. SEM images of the samples printed using CX-Wood.

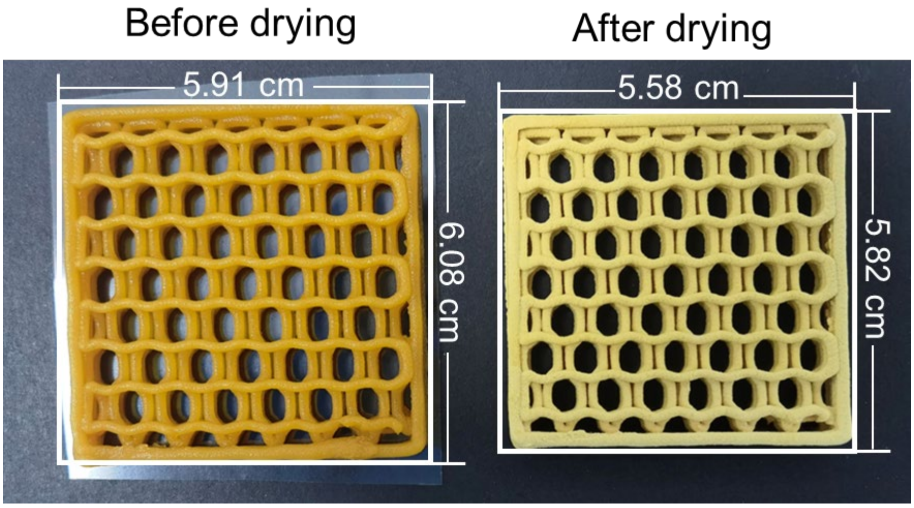

Supplementary Fig. 27. Comparison of samples printed with CX-Wood before and after drying.

Supplementary Discussion

The mechanical properties of CX-Wood

Supplementary Fig. 28-31 shows the mechanical properties of CX-Wood. The tensile strength and Young's modulus of CX-Wood were 12.5 MPa and 151.0 MPa, respectively, while its compressive strength and compressive modulus were 8.7 MPa and 83.0 MPa (**Supplementary Fig. 28**). Additionally, we compared the mechanical strength between CX-Wood and natural woods (basswood and balsawood). Due to the anisotropic nature of natural woods[21], they exhibit different tensile strengths in the "T-the radial direction of the wood", "R-the tangential direction of the wood", and "L-the longitudinal direction of the wood" (**Supplementary Fig. 28a**). Natural basswood exhibits strong tensile performance only in the "L" direction, with tensile strength and Young's modulus of 70.6 MPa and 547.3 MPa, respectively. However, its tensile properties in both the T- and R-directions are inferior to those of CX-Wood (**Supplementary Fig. 28b**). Natural balsa wood, in all three directions (T, R, and L), demonstrates lower tensile performance than CX-Wood (**Supplementary Fig. 28b**).

Regarding compressive strength, both basswood and balsawood demonstrate strong compressive performance in the "TR" direction (**Supplementary Fig. 28c**). Under a pressure of 2000 N, basswood exhibits compressive strength and compressive modulus of 8.4 MPa and 169.0 MPa respectively in the "TR" direction, while balsawood shows values of 6.1 MPa and 126.3MPa in the same direction. However, the compressive properties of both woods in the "LR" direction are inferior to those of CX-Wood (**Supplementary Fig. 28c**).

In addition, dispersion of CX-Wood in an aqueous solution of polyurethane could be used as inks for direct printing. The as-printed structure was then dried by heating. Meanwhile, the post-crosslinking reaction between CX-Wood and polyurethane occurred during the process. The as-obtained structure exhibited good flexibility and retained its integrity after five compression cycles, whereas natural wood underwent structural collapse after just a single compression (**Supplementary Fig. 29**).

Encouraged by this result, a series of 3D shapes were printed using CX-Wood. To further demonstrate the advantages, structures printed from CX-Wood and commercially available carboxymethyl cellulose (CMC) were evaluated. Due to the presence of lignin in CX-Wood, which provides adhesive and structural support, the printed samples were self-standing, whereas the structures printed using commercial carboxymethyl cellulose were prone to collapse (**Supplementary Fig. 30**)[22]. Additionally, the tensile strength of the printed structures from commercial CMC are also inferior to those of CX-Wood (**Supplementary Fig. 31**). This is attributed to its structural heterogeneity, relatively thin pore walls, and the presence of cracks in certain areas compared to the internal structure of CX-Wood (**Supplementary Fig. 32**).

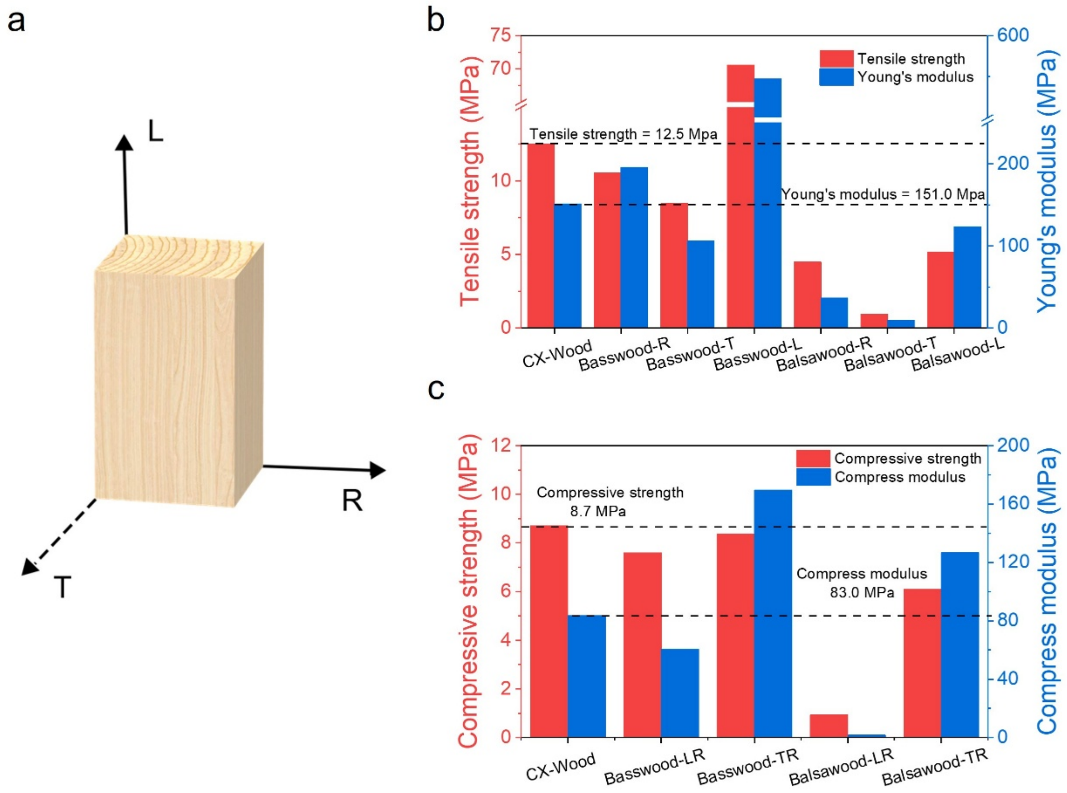

Supplementary Fig. 28. (a) Schematic diagram of Tensile and compression testing directions for natural wood (basswood and balsawood). (b) Tensile (tensile strength and Young's modulus) properties of CX-Wood and basswood and balsawood (The data are from single measurement). (c) Compressive (compress strength and compress modulus) properties of CX-Wood and basswood and balsawood (the pressure is 2000 N) (The data are from single measurement).

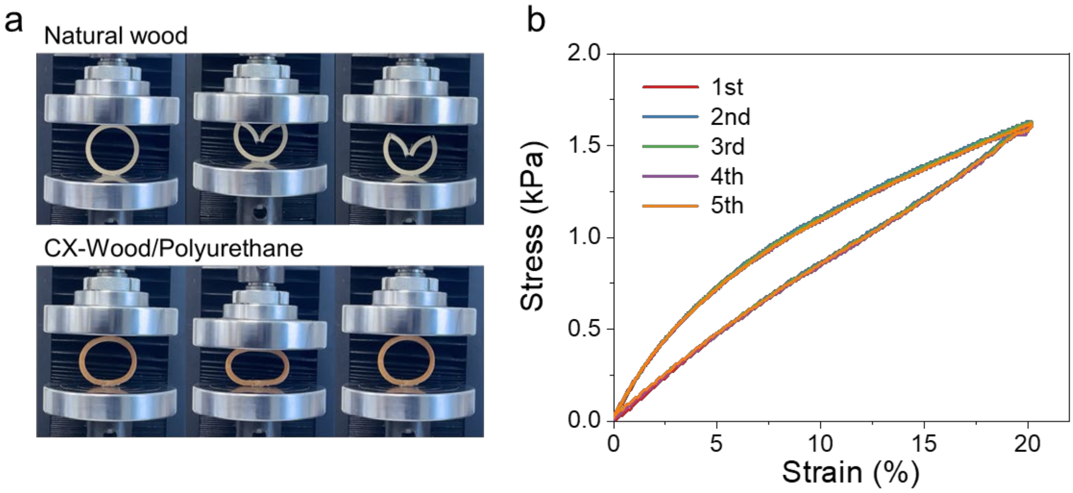

Supplementary Fig. 29. (a) Compression images of natural wood and CX-Wood/Polyurethane. (b) Five-cycle compression curves of CX-Wood/Polyurethane.

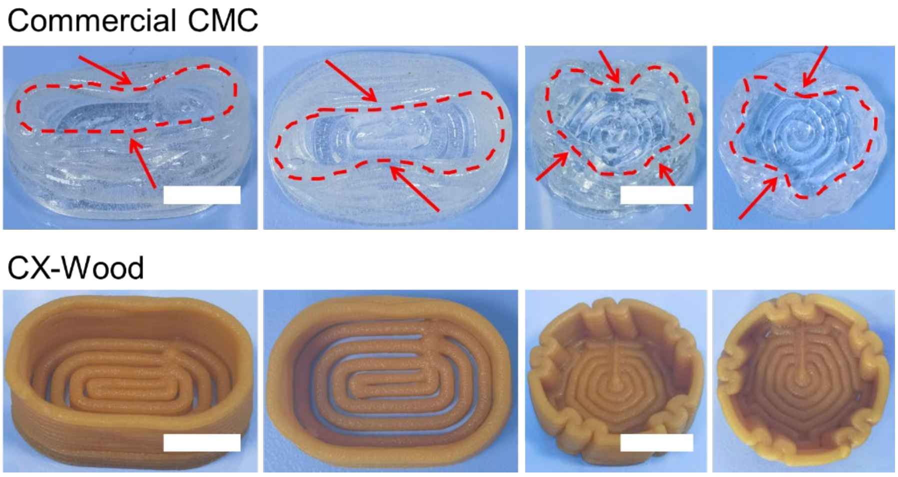

Supplementary Fig. 30. Samples printed using commercial CMC and CX-Wood. As shown in the figure, the samples printed with CX-Wood retain their structural integrity, while those printed with commercial CMC exhibit varying degrees of collapse (highlighted in red), scale bar = 2 cm.

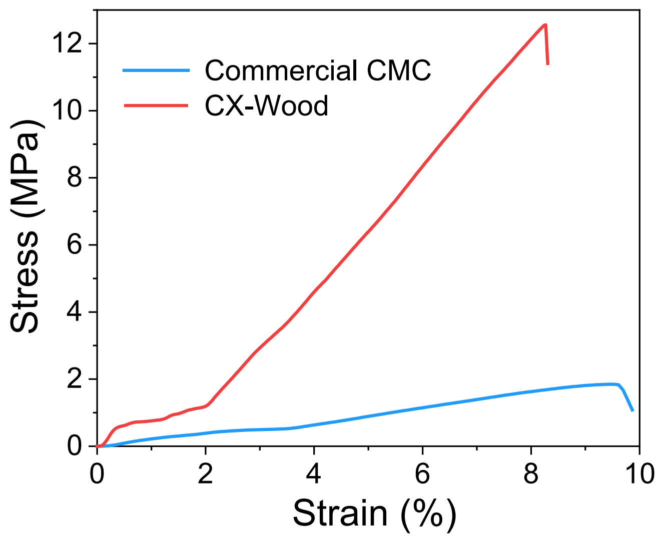

Supplementary Fig. 31. Tensile strength of the structure printed from commercial CMC and CX-Wood.

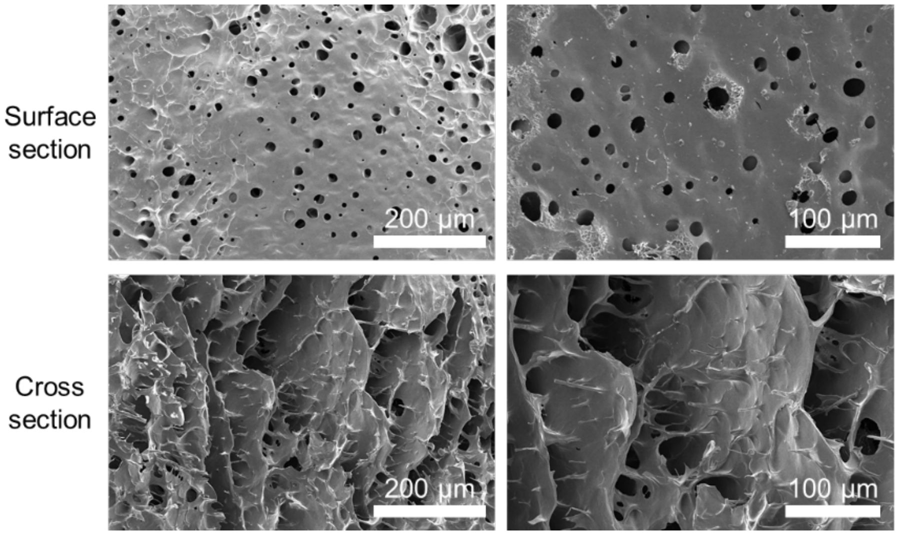

Supplementary Fig. 32. SEM images of the samples printed using commercial CMC.

The flame retardant properties of CX-Wood

Supplementary Fig. 33-35 shows the flame retardancy of CX-Wood. The thermogravimetric analysis (TG) and differential thermogravimetric analysis (DTG) curves in the continuous and trigger modes indicate that CX-Wood generated more residues than natural wood (**Supplementary Fig. 33**).

The enhanced flame retardancy stems from a dual mechanism. On one hand, the abundant internal mesoporous structure of CX-Wood exhibits exceptional thermal insulation properties, effectively blocking the transfer of combustion heat to the material's interior and preventing deep-seated combustion. Simultaneously, the small pore size suppresses gas flow, reducing oxygen penetration into the combustion zone and directly weakening the combustion intensity[23]. On the other hand, during combustion, CX-Wood undergoes carbonization to form a dense and stable char layer that acts as a barrier between the atmosphere and the material's internal structure, thereby suppressing flame propagation[24].

The maximum LOI value of CX-Wood was 39.5%, which is higher than that (19.8%) of natural wood (**Supplementary Fig. 34**). Vertical burning tests indicated that the natural wood was flammable and there was no rating according to the UL-94 standard. In comparison, CX-Wood exhibited superior self-extinguishing performance after two ignitions (fire treatment for 10 s each time), reaching the highest level (V-0 grade, UL-94 standard) (**Supplementary Fig. 35**)[25]. The CONE results indicated that the values of heat-release rate, total heat release, and the total smoke production for CX-Wood were significantly lower than natural wood. The residual char of CX-Wood reached 39.7% at the end of the CONE tests, and there was almost no residue for natural wood (**Supplementary Fig. 36**). Further analysis of the carbon residue results from CC tests of natural wood and CX-Wood revealed that the alkaline environment induced by Na^+ in CX-Wood facilitated dehydration reactions, enabling rapid formation of continuously growing carbon layers and thereby enhancing its flame retardant nature (**Supplementary Fig. 36**). The synergistic effect between "porous structure" and "post-

combustion char layer" of CX-Wood enhances its flame retardancy.

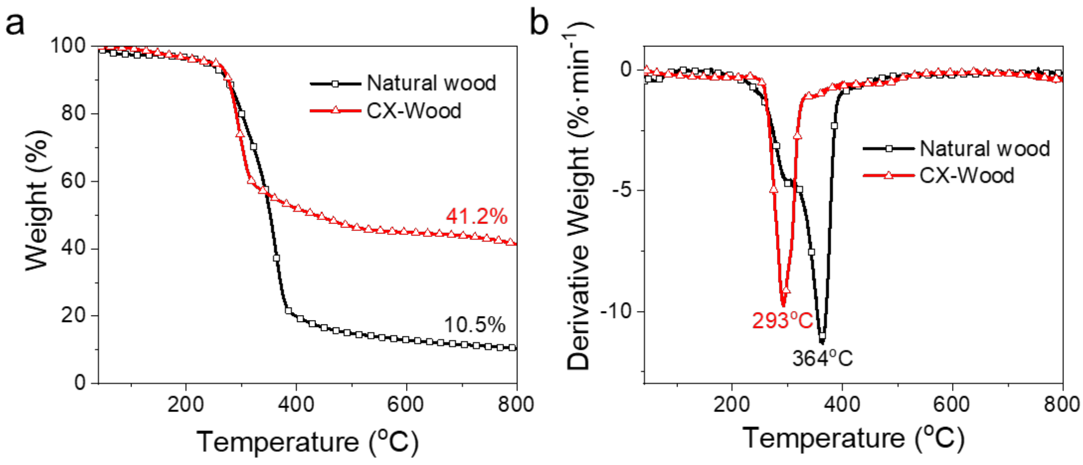

Supplementary Fig. 33. (a) TG and (b) DTG curves for Natural wood and CX-Wood in a nitrogen atmosphere.

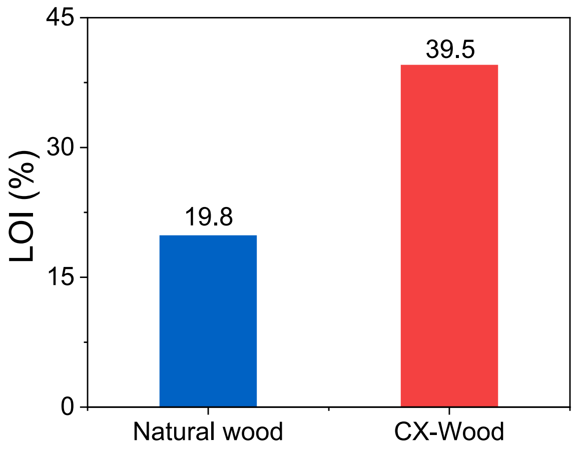

Supplementary Fig. 34. The limiting oxygen index of Natural wood and CX-Wood (The data are from single measurement).

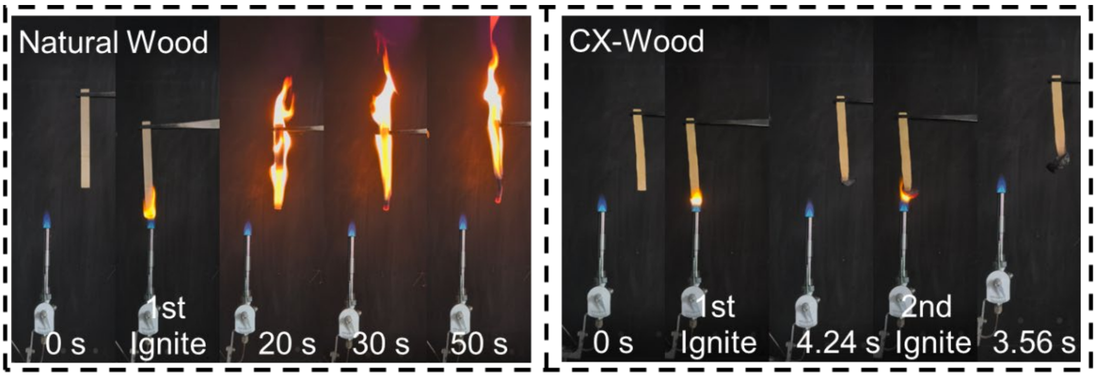

Supplementary Fig. 35. Vertical burning of Natural wood and CX-Wood (UL-94 test).

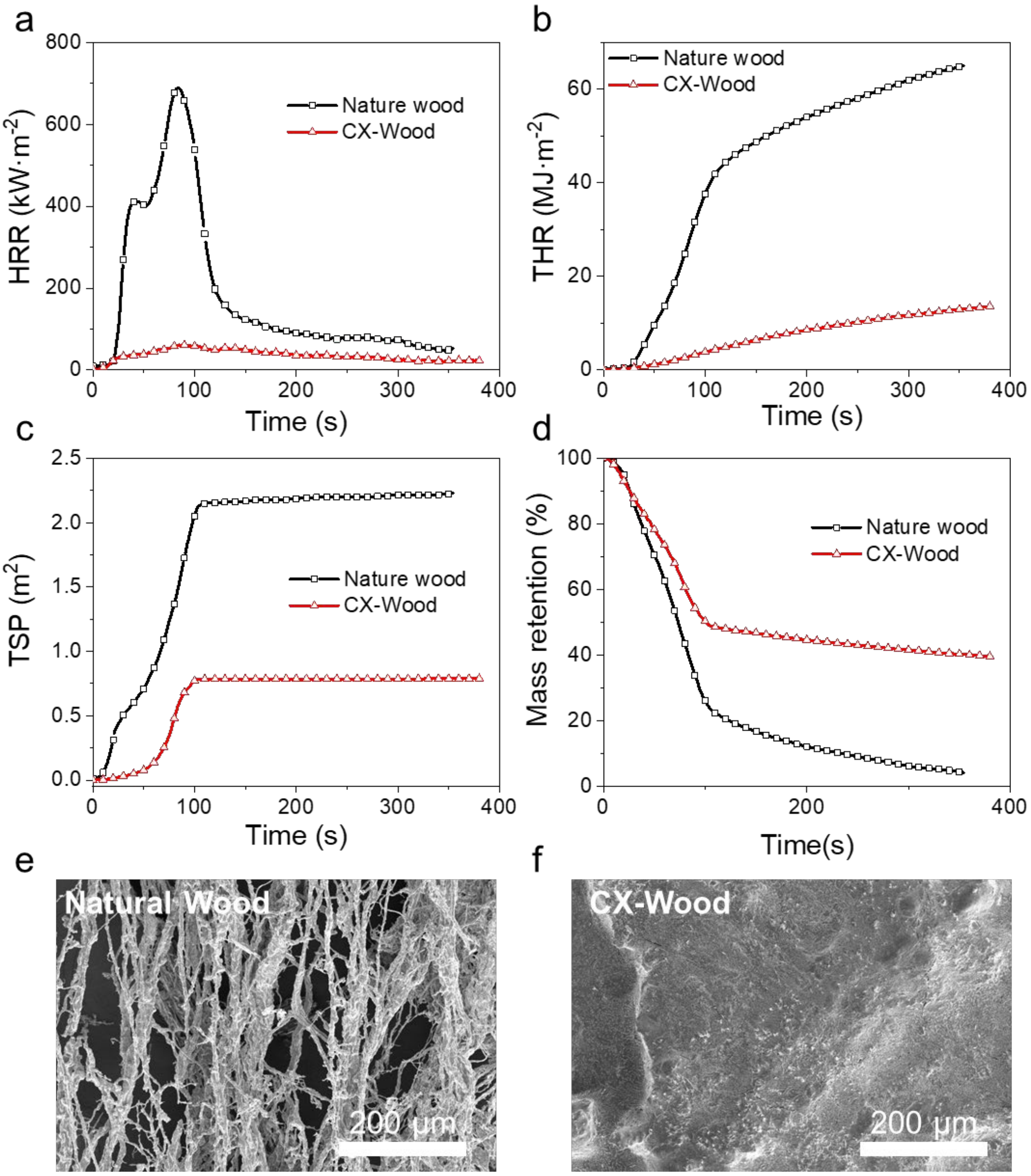

Supplementary Fig. 36. Cone calorimeter of Natural wood and CX-Wood. (a) HRR curves. (b) THR curves. (c) TSP curves. (d) Mass retention curves. (e) SEM images of the residual char of Natural wood. (f) SEM images of the residual char of CX-Wood.

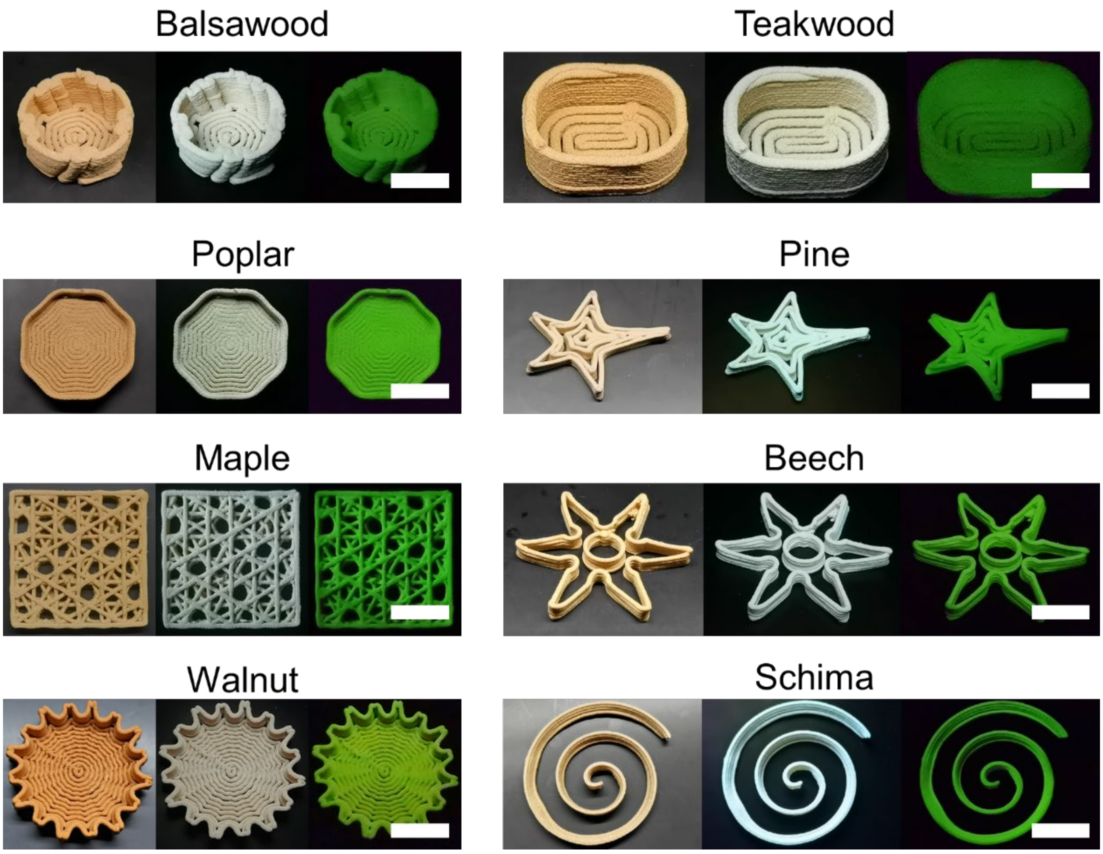

Supplementary Fig. 37. Printed shapes using CX-Wood made from different wood species, scale bar = 2 cm.

Supplementary Movie 1. The 3D printing of CX-Wood using direct ink writing.

Supplementary References

1. Kono H, Oshima K, Hashimoto H, Shimizu Y, Tajima K. NMR characterization of sodium carboxymethyl cellulose: Substituent distribution and mole fraction of monomers in the polymer chains. *Carbohyd. Polym.* **146**, 1-9 (2016).
2. Frisch, M.J., Trucks, G.W., *et al.* (2016) Gaussian 16 Revision A.0.
3. Becke A.D. Density-functional exchange-energy approximation with correct asymptotic behavior. *Phys. Rev. Appl.* **38**, 3098 (1988).
4. Grimme S, Antony J, Ehrlich S, Krieg H. A consistent and accurate ab initio parametrization of density functional dispersion correction (DFT-D) for the 94 elements H-Pu. *J. Chem. Phys.* **132**, 154104. (2010).
5. Jensen F, *et al.* Polarization consistent basis sets: Principles. *J. Chem. Phys.* **115**, 9113-9125 (2001).
6. Lu T, *et al.* A comprehensive electron wavefunction analysis toolbox for chemists, Multiwfn. *J. Chem. Phys.* **161**, 082503 (2024).
7. Humphrey W, Dalke A, Schulten K. VMD: visual molecular dynamics. *J. Mol. Graph.* **14**, 33-38 (1996).
8. Neese F, Wennmohs F, Becker U, Riplinger C. The ORCA quantum chemistry program package. *J. Chem. Phys.* **152**, 224108 (2020).
9. Adamo C, Barone V. Toward reliable density functional methods without adjustable pa rameters: The PBE0 model. *J. Chem. Phys.* **110**, 6158-6170 (1999).
10. Weigend F, Ahlrichs R. Balanced basis sets of split valence, triple zeta valence and quadruple zeta valence quality for H to Rn: Design and assessment of accuracy. *Phys. Chem. Chem. Phys.* **7**, 3297-3305 (2005).
11. VandeVondele J, Krack M, Mohamed F, Parrinello M, Chassaing T, Hutter J. Quickstep: fast and accurate density functional calculations using a mixed Gaussian and plane waves approach. *Comput. Phys. Commun.* **167**, 103-128 (2005).
12. Grimme S, Bannwarth C, Shushkov P. A robust and accurate tight-binding quantum chemical method for structures, vibrational frequencies, and noncovalent interactions of large molecular systems parametrized for all spd-block elements (Z = 1-86). *J. Chem. Theory Comput.* **13**, 1989-2009 (2017).
13. Perdew JP, Burke K, Ernzerhof M. Generalized gradient approximation made simple. *Physical review letters.* **77**, 3865-3868 (1996).
14. Dunning TH, Hay PJ. Gaussian basis sets for molecular calculations. *Methods of Electronic Structure Theory.* **3**, 1-27 (1997).
15. Lefebvre C, Rubez G, *et al.* Accurately extracting the signature of intermolecular interactions present in the NCI plot of the reduced density gradient versus electron density. *Phys. Chem. Chem. Phys.* **19**, 17928-17936 (2017).
16. Lu T, Chen FW. Multiwfn: A multifunctional wavefunction analyzer. *J. Comput.Chem.* **33,** 580-592 (2012).
17. Thakur MSH, *et al.* Three-dimensional printing of wood. *Sci. Adv.* **10**, eadk3250 (2024).
18. Wu TT, *et al.* Deep eutectic solvent-assisted 3D printing of lignocellulosic biomass. *Chem. Eng. J.* **523**, 168671 (2025).

19. Niu Y, *et al.* A universal strategy for achieving dual cross-linked networks to obtain ultralong polymeric room temperature phosphorescence. *Sci. China. Chem.* **66**, 1161-1168 (2023).
20. Yin WM , *et al.* Up-recycling of waste wood into value-added room temperature phosphorescent materials. *Nat. Commun.* **16**, 7978 (2025).
21. Chen C, *et al.* Structure–property–function relationships of natural and engineered wood. *Nat Rev Mater.* **5**, 642-666 (2020).
22. Xia Q, *et al.* In Situ Lignin Modification toward Photonic Wood. *Adv. Mater.* **33**, 2001588 (2021).
23. Wang X, *et al.* Polyimide-Coating-on-Aramid nanofiber strategy toward ultralight organic aerogels with multifunctional properties. Chem. Eng. J. **499**, 155939 (2024).
24. Shi R, *et al.* Influence of Na^{+} and Ca^{2+} on flame retardancy, thermal degradation, and pyrolysis behavior of cellulose fibers. *Carbohyd. Polym.* **157**, 1594-1603 (2017).
25. Wang X, et al. Carbon-family materials for flame retardant polymeric materials. Prog.Polym. *Sci.* **69**, 22-46 (2017).
