# Supplementary material for: 3D-printable phosphorescent woody materials
Source: Nat Commun. 2026 Mar 11;17:3796. doi: 10.1038/s41467-026-70488-y (PMC13111626; doi:10.1038/s41467-026-70488-y)
Supplement: Supplementary file 2 — Description of Additional Supplementary Files [file 41467_2026_70488_MOESM2_ESM.pdf]

Description of Additional Supplementary Files

File Name: Supplementary Movie 1
Description: The 3D printing of CX-Wood using direct ink writing.

File Name: Supplementary Data 1
Description: Atomic coordinates of optimised models.
